# Stacking disease resistance and mineral biofortification in cassava varieties to enhance yields and consumer health

**DOI:** 10.1111/pbi.13511

**Published:** 2020-12-10

**Authors:** Narayanan Narayanan, Getu Beyene, Raj Deepika Chauhan, Michael A. Grusak, Nigel J. Taylor

**Affiliations:** ^1^ Donald Danforth Plant Science Center St. Louis MO USA; ^2^ USDA‐ARS Edward T. Schafer Agricultural Research Center Fargo ND USA; ^3^ Present address: Pairwise Durham NC USA

**Keywords:** cassava, cassava brown streak disease, cassava mosaic disease, RNAi, biofortification, iron, zinc, trait stacking

## Abstract

Delivering the benefits of agricultural biotechnology to smallholder farmers requires that resources be directed towards staple food crops. To achieve effect at scale, beneficial traits must be integrated into multiple, elite farmer‐preferred varieties with relevance across geographical regions. The staple root crop cassava (*Manihot esculenta*) is consumed for dietary calories by more than 800 million people, but its tuberous roots provide insufficient iron and zinc to meet nutritional needs. In Africa, cassava yields are furthermore limited by the virus diseases, cassava mosaic disease (CMD) and cassava brown streak disease (CBSD). In this study, we strove to develop cassava displaying high‐level resistance to CBSD and CMD to attain food and economic security for cassava farmers, along with biofortified levels of iron and zinc to enhance consumer health. RNAi‐mediated technology was used to achieve resistance to CBSD in two East African and one Nigerian farmer‐preferred cultivars that harboured resistance to CMD. The Nigerian cvs. TMS 95/0505 and TMS 91/02324 were modified with T‐DNA imparting resistance to CBSD, along with *AtIRT1* (major iron transporter) and *AtFER1* (ferritin) transgenes to achieve nutritionally significant levels of iron and zinc in cassava storage roots (145 and 40 µg/g dry weight, respectively). The inherent resistance to CMD was maintained in all four disease resistant and mineral enhanced cassava cultivars described here, demonstrating that this technique could be deployed across multiple farmer‐preferred varieties to benefit the food and nutritional security of consumers in Africa.

## Introduction

Cassava (*Manihot esculenta Crantz*) provides dietary calories for more than half a billion people worldwide and is a major staple food crop in sub‐Saharan Africa. The two viral diseases cassava mosaic disease (CMD) and cassava brown streak disease (CBSD) present serious constraints to cassava production in Africa. Plants affected by CBSD develop necrotic lesions within their storage roots, rendering them inedible and without value as food or feed. CMD compromises the plant’s photosynthetic capacity resulting in reduced storage root yields. CBSD alone has been estimated to result in annual losses to farmers of US$750 million across Kenya, Tanzania, Uganda and Malawi (Patil *et al*., 2015; Tomlinson *et al*., 2018). Originally confined to East Africa, CBSD is spreading through central Africa towards West Africa, where it presents a risk to food and economic security for hundreds of millions of people, especially in Nigeria, the world’s largest cassava‐producing country (FAO, 2019; Otekunrin and Sawicka, 2019).

Cassava brown streak disease is caused by the closely related, single stranded RNA viruses *Cassava brown streak virus* (CBSV) and *Ugandan cassava brown streak virus* (UCBSV), family Potyviridae, genus Ipomovirus (Winter *et al*., 2010). No CBSD‐resistant cassava varieties are presently available to farmers. CMD is caused by cassava mosaic geminiviruses belonging to the family Geminiviridae, genus Begomovirus (Patil and Fauquet, 2009). Like CBSD, CMD is transmitted by the whitefly *Bemisia tabaci*, and spread by farmers who plant infected stem cuttings used to establish subsequent cropping cycles (Hillocks and Thresh, 2000; Hillocks et al., 1996). Transgenic expression of a hairpin RNAi construct derived from fused, near full‐length coat protein (CP) sequences of UCBSV and CBSV was shown to provide high‐level resistance to CBSD in cassava cultivar TME 204 in the greenhouse (Beyene *et al*., 2017a) and across multi‐year, multi‐location field trials in Uganda and Kenya (Wagaba *et al*., 2016). An unexpected result of producing RNAi‐mediated CBSD resistance in TME 204 was a concomitant loss of inherent resistance to CMD across all 30 independent transgenic lines tested (Beyene *et al*., 2016). Loss of CMD resistance in TME 204 and in plants of cultivars TME 7, TME 3, TME 14 and TME 419 was found to occur during the morphogenic processes used to generate transgenic cassava plants (Beyene *et al*., 2016; Chauhan and Taylor, 2018). Due to the endemic nature of CMD in Africa, all cassava varieties must exhibit robust resistance to infection by geminiviruses. Overcoming or circumventing loss of resistance to CMD is therefore critical if the value of RNAi‐mediated CBSD resistance and other beneficial transgenic and gene edited technologies are to be delivered to cassava farmers in sub‐Saharan Africa.

Micronutrient malnutrition affects more than one‐half of the world’s population and especially impairs the wellbeing of women and preschool children (SCN, 2004). The ‘hidden hunger’ posed by micronutrient deficiencies represents a significant threat to human health, particularly in regions that rely on starchy staple crops such as cassava (Gegios *et al*., 2010). In Nigeria, 39 per cent of children (0–19 years) are anaemic due in large part to iron deficiency, and 63 per cent of children (0–5 years) suffer from zinc deficiency (Harika *et al*., 2017). Scientific evidence and predictive cost‐benefit analyses identifies biofortification of staple food crops as a reliable strategy for delivering elevated micronutrients to vulnerable populations. Although an excellent source of starch, cassava storage roots provide less than 10% and 15% of the estimated average requirements (EAR) for iron and zinc respectively (Hefferon, 2015; Zimmermann and Hurrell, 2007). Efforts to enhance iron and zinc concentrations of cassava storage roots through traditional breeding are constrained by insufficient genetic variation for these traits within existing germplasm. Metabolic engineering therefore offers unique potential for increasing genetic variation of storage root micronutrient concentrations. We recently demonstrated that storage roots produced by field‐grown cassava plants co‐expressing *AtIRT1* (major iron transporter) and *AtFER1* (storage protein) accumulated significantly increased concentrations of iron and zinc (Narayanan *et al*., 2019). Elevated mineral levels within the biofortified storage roots and their processed products were determined to be nutritionally available at EAR values of 40%–50% iron and 60%–70% zinc, levels that could have a significant impact on the health of children and women in West Africa (Narayanan *et al*., 2019).

In this study, we report development of RNAi‐mediated CBSD resistance in four African farmer‐preferred cassava cultivars without compromising resistance to CMD. In addition, CBSD and CMD resistance traits were stacked with mineral biofortification traits in two Nigerian cassava cultivars, to generate products enhanced to meet the nutritional needs of smallholder farmers and consumers in East and West Africa.

## Results

### Production and molecular analysis of transgenic plants

The RNAi inverted repeat construct p5001 (Beyene *et al*., 2017a) was transformed into CMD resistant farmer‐preferred East African cassava cultivars NASE 13 and NASE 14 and the Nigerian cultivar TMS 98/0505. An additional construct p9001 (Figure S1) was generated in which the CBSV and UCBSV‐CP‐derived, inverted repeat sequence present in p5001 was stacked with the *Arabidopsis* iron transporter (*AtIRT1*) and storage protein ferritin (*AtFER1*) (Narayanan *et al*., 2019). p9001 was used to genetically transform Nigerian cultivars TMS 98/0505 and TMS 91/02324. Regenerated plants were analysed to confirm presence and mRNA expression of transgenes imparting the traits of interest (Table S1). In the case of p5001, 77% of NASE 13 and 95% of TMS 98/0505 plant lines were confirmed as transgenic for the presence of the CBSV/UCBSV‐CP‐derived inverted repeat cassette. In contrast, 37% of regenerated NASE 14 plant lines were found to be PCR‐positive for p5001, reflecting the relatively recalcitrant nature of this cultivar for the transformation system. Between 47% and 50% of regenerated plants of TMS 98/0505 and TMS 91/02324 were PCR‐positive for the presence of all three expression cassettes in p9001 (CP‐inverted repeat, *IRT1* and *FER1*) in the two cultivars, respectively (Table S1). mRNA expression of the *IRT1* and *FER1* transgenes was confirmed by RT–qPCR in p9001 transgenic events (Figure S2). Southern blotting performed on PCR‐confirmed p5001 and p9001 transgenic events revealed that 40%–87% of plant lines carried 1‐2T‐DNA copies across the four cultivars (Figure S3).

**Figure 1:**
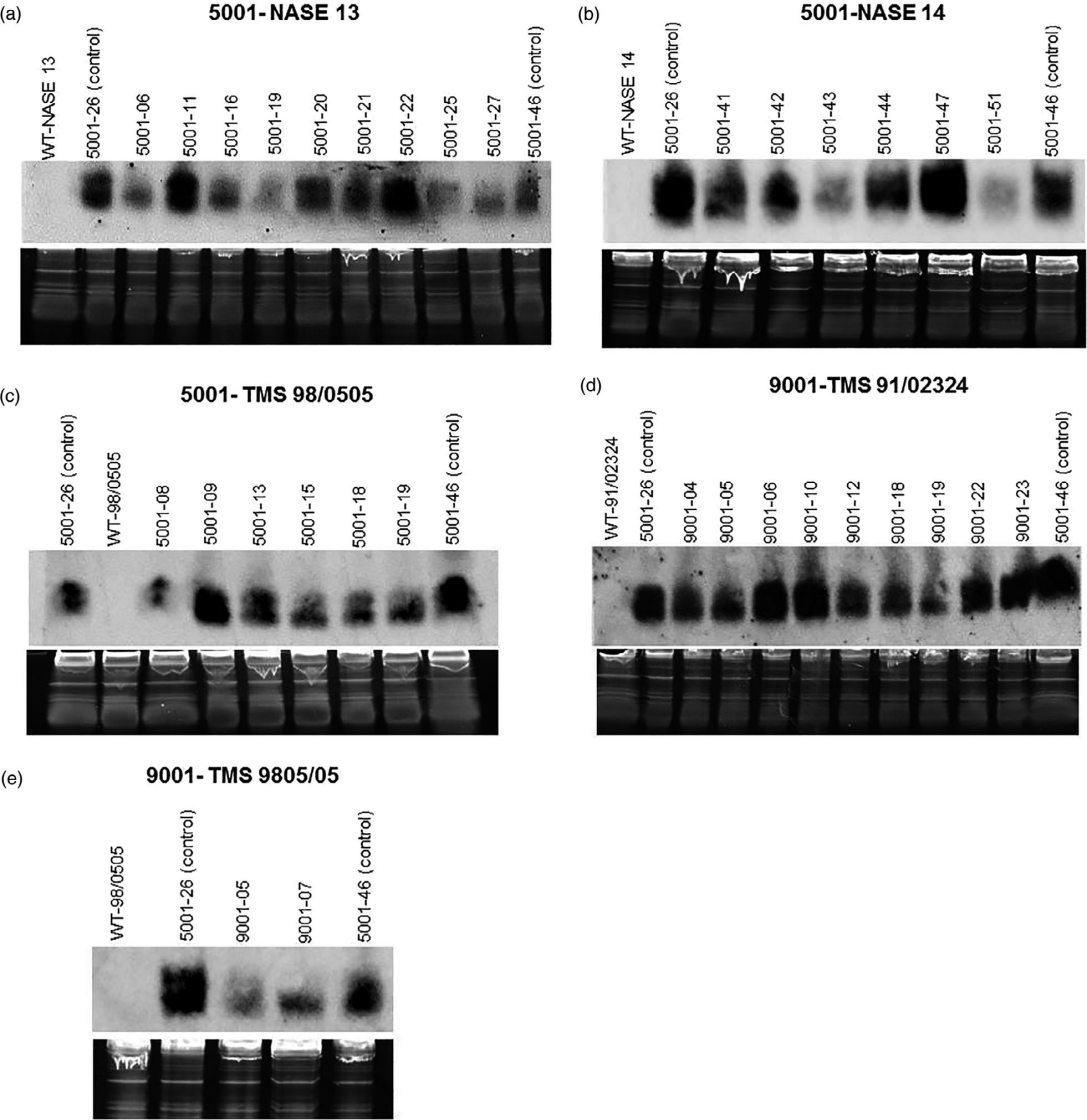
Detection of siRNAs derived from coat protein (CP) sequences of *Cassava brown streak virus* and *Ugandan cassava brown streak* virus in cassava cultivars (a) 5001‐NASE 13, (b) 5001‐NASE 14, (c) 5001‐TMS 98/0505, (d) 9001‐TMS 91/02324 and (e) 9001‐TMS 98/0505. Transgenic plants carrying p5001 or p9001 harbouring inverted repeats of the CP genes derived from CBSV and UCBSV were detected with probe specific to CP of CBSV. RNA was extracted from leaves of in vitro plantlets. Transgenic plant lines 5001‐26 and 5001‐46 regenerated from cultivar TME 204 were used as positive controls. WT‐wild type.

Accumulation of CP‐derived siRNAs was determined by Northern blots performed on 32 independent plant lines of NASE 13, NASE 14, TMS 95/0505 and TMS 91/02324 transgenic for p5001 or p9001 (Figure 1). All blots included positive control siRNA extracted from plants of TME 204 events 5001‐26 and 5001‐46 grown under the same conditions. Events 5001‐26 and 5001‐46 were shown previously to accumulate high levels of siRNAs (Beyene *et al*., 2017a) and to display resistance to CBSD under field trials in East Africa (Wagaba *et al*., 2016). All transgenic plants were seen to accumulate detectable CP‐derived siRNAs but did so at variable levels. Seven out of 30 lines showed medium levels of signal intensity (30%–60% of the positive control), 16 lines had high signal intensities (60%–100% of positive control), and the remaining seven lines had very high signals (two in NASE 13 (Figure 1a), one each in NASE 14 (Figure 1b) and TMS 98/0505 (Figures 1c, e) and three in TMS 91/02324 (Figure 1d) at equal or greater than the TME 204 controls (100%–140% of positive control) (Figure 1).

### p5001 and p9001 transgenic plants show resistance to challenge with CBSV

**Figure 2:**
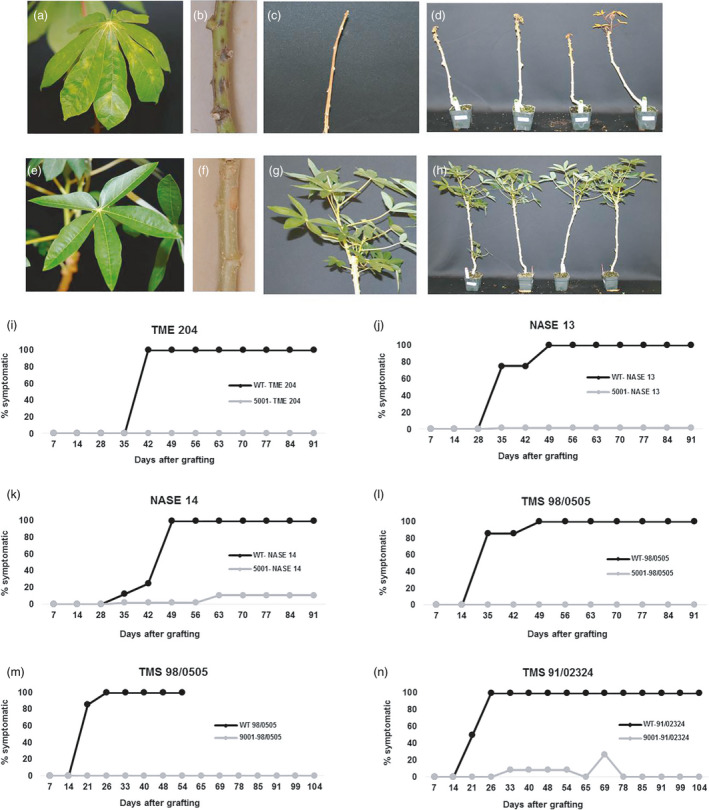
CBSD symptoms in leaves and stems of (a‐d) non‐transgenic wild‐type TMS 98/0505 and (e‐h) transgenic p9001‐TMS 98/0505 plants. All plants were bud graft inoculated with the CBSV isolate Naliendele (CBSV [TZ:Nal3‐1:07] under greenhouse conditions. Line graph showing disease progression over time as per cent plants showing CBSD symptoms (i) 5001‐TME 204, (j) 5001‐NASE 13, (k) 5001‐NASE 14, (l) 5001‐TMS 98/0505, (m) 9001‐TMS 98/0505 and (n) 9001‐TMS 91/02324 transgenic and respective wild‐type plants. Six to ten plants were challenged per cultivar/line, and experiments were repeated at least twice.

Transgenic plants expressing medium to very high levels of siRNA accumulation were established in the greenhouse and bud graft inoculated with the Naliendele strain of CBSV (Wagaba *et al*., 2013). Non‐transgenic plants displayed first CBSD leaf symptoms 2‐3 weeks after inoculation, becoming apparent as small chlorotic spots on the leaf lamina (Figure 2a), with subsequent development of brown lesions on stems (Figure 2b). By 6–7 weeks after inoculation, 80‐100% of non‐transgenic wild‐type controls for all four cultivars exhibited leaf and stem CBSD symptoms. Severity of response was most dramatic in cv. TMS 98/0505. Non‐transgenic controls of this cultivar developed shoot necrosis on young growth, followed by die‐back from the apex downwards, progressing to complete death of the plant 5–7 weeks after bud grafting (Figures 2c‐d). In contrast, no CBSD symptoms were observed on shoot tissues of NASE 13, NASE 14 or TMS 98/0505 transgenic for construct p5001 or on TMS 98/0505 and TMS 91/02324 transgenic for construct p9001 (Figures 2e‐h; j‐n). As expected, plants of the TME 204 positive controls 5001‐26 and 5001‐46 also remained asymptomatic on leaves and stems over this period (Figure 2i).

**Figure 3:**
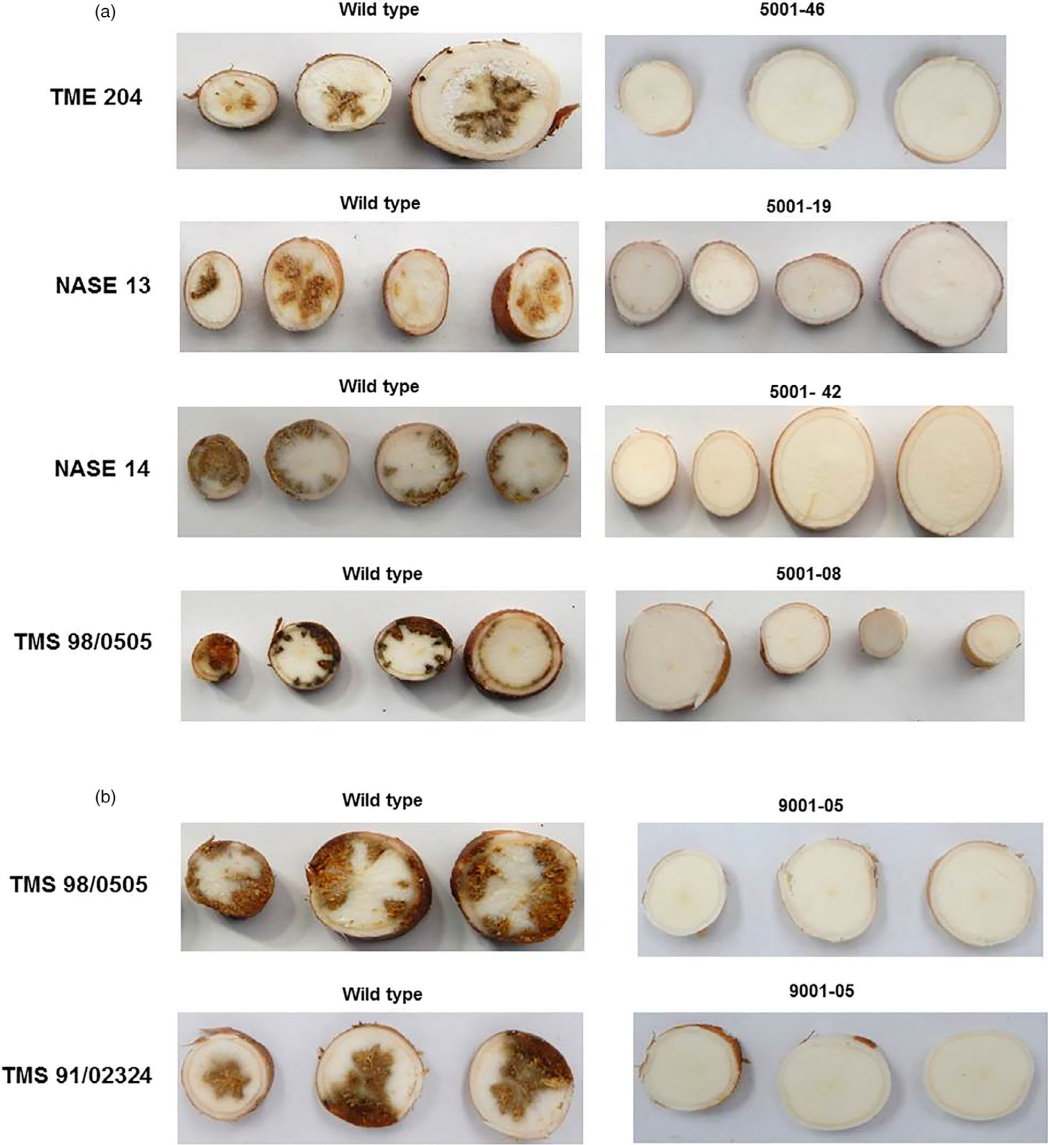
CBSD symptoms within storage roots of (a) transgenic p5001 and (b) p9001 plant lines with respective non‐transgenic wild‐type control lines. Representative storage root slices were taken 12–14 weeks after graft inoculation with CBSV isolate Naliendele (CBSV [TZ:Nal3‐1:07] under greenhouse conditions. Non‐transgenic wild‐type storage roots show presence of severe brown necrotic symptoms typical of CBSD. Storage roots harvested from p5001 and p9001 transgenic lines remained free of CBSD symptoms. Experiments were repeated at least twice.

Storage roots were harvested 12–14 weeks after bud graft inoculation and sliced transversely along their length to allow visual evaluation for CBSD symptom incidence and severity using a 1–5 scale (Figure 3). All non‐transgenic controls displayed CBSD symptoms within storage roots at 70%–100% incidence (Figure 3) with average symptom severities of 1.6–4.6 (Table 1). Storage roots from 9/9 events of p5001 in NASE 13, 6/6 events of p5001 in NASE 14 and 5/6 events of p5001 in TMS 98/0505 were found to be free of CBSD symptoms. Likewise, roots of both lines of p9001 in TMS 98/0505 and 7/9 events of p9001 in TMS 91/02324 remained asymptomatic (Table 1). Among 32 transgenic lines tested from the four cultivars, more than 97% (189 out of 195 total plants examined) were free of CBSD symptoms in their storage roots (Table 1). In the six lines positive for the presence of CBSD, storage root symptoms were very mild, with average scores of 1.23 (Table 1). Expression of CP‐derived siRNA was positively and strongly correlated with levels of resistance to CBSD tested in greenhouse conditions (Fig. 1c, d). Positive control TME 204 transgenic events 5001‐26 and 5001‐46 showed no CBSD symptoms in storage roots, in a manner similar to that described earlier (Wagaba *et al*., 2016).

**Table 1:** Number of plants and storage roots showing CBSD symptoms and average symptom severity (scale 1‐5)^†^

	# Of plants challenged	# Of symptomatic plants	# Of roots harvested	# Of symptomatic roots	Average symptom severity (scale 1‐5)
WT‐TME 204	6	6	27	16	1.61 ± 0.71
5001‐T0426	6	0	32	0	1
5001‐T0446	6	0	24	0	1
WT‐NASE 13	6	6	21	15	2.11 ± 1.09
5001‐N13006	6	0	12	0	1
5001‐N13011	6	0	16	0	1
5001‐N13016	6	0	20	0	1
5001‐N13019	6	0	20	0	1
5001‐N13020	6	0	19	0	1
5001‐N13021	6	0	20	0	1
5001‐N13022	5	0	17	0	1
5001‐N13025	8	0	31	0	1
5001‐N13027	9	0	31	0	1
WT‐NASE 14	6	6	10	10	3.12 ± 0.26
5001‐N14041	6	0	13	0	1
5001‐N14042	6	0	23	0	1
5001‐N14043	6	0	17	0	1
5001‐N14044	6	0	15	0	1
5001‐N14047	6	0	12	0	1
5001‐N14051	6	0	16	0	1
WT‐98/0505	12	12	39	39	4.19 ± 0.70
5001‐985008	6	1	18	1	1.04 ± 0.05
5001‐985009	6	0	21	0	1
5001‐985013	6	0	23	0	1
5001‐985015	6	0	28	0	1
5001‐985018	6	0	25	0	1
5001‐985019	6	0	26	0	1
9001‐985005	6	0	20	0	1
9001‐985007	9	0	7	0	1
WT‐91/02324	6	6	26	25	4.6 ± 0.46
9001‐914004	6	0	29	0	1
9001‐914005	7	0	24	0	1
9001‐914006	7	0	30	0	1
9001‐914010	5	0	21	0	1
9001‐914012	6	0	24	0	1
9001‐914018	7	2	26	2	1.17 ± 0.72
9001‐914019	7	3	29	3	1.23 ± 0.84
9001‐914022	6	0	21	0	1
9001‐914023	5	0	18	0	1

^†^ Bud graft inoculated plants were harvested between 12 and 14 weeks growth in the greenhouse. Shoots and storage roots were assessed for presence of CBSD symptoms. Severity of CBSD symptoms was scored using a 1‐5 scale as per Hillocks and Thresh (2000) where 1 = no symptoms and 5 = maximum symptoms.

### Detection of CBSV virus in transgenic plants

At harvest, storage roots were sampled from plants challenged with CBSV and from non‐infected self‐grafts. Three or four clonal plants per transgenic line were assayed by qRT‐PCR to detect presence of CBSV. All plants of non‐transgenic controls showed presence of CBSV virus, while all plants of asymptomatic transgenic p5001 and p9001 plants had no detectable CBSV within their storage root tissues (Figure S4). Transgenic lines showing mild CBSD symptoms in the storage roots also accumulated low levels of CP‐derived siRNAs (lines 5001‐08, Fig. 1c; 9001‐19, Fig. 1d & Table 1) and very low detectable levels of CBSV in their storage roots (Figure S4d,f).

### p5001 and p9001 transgenic plants show resistance to CMD

**Figure 4:**
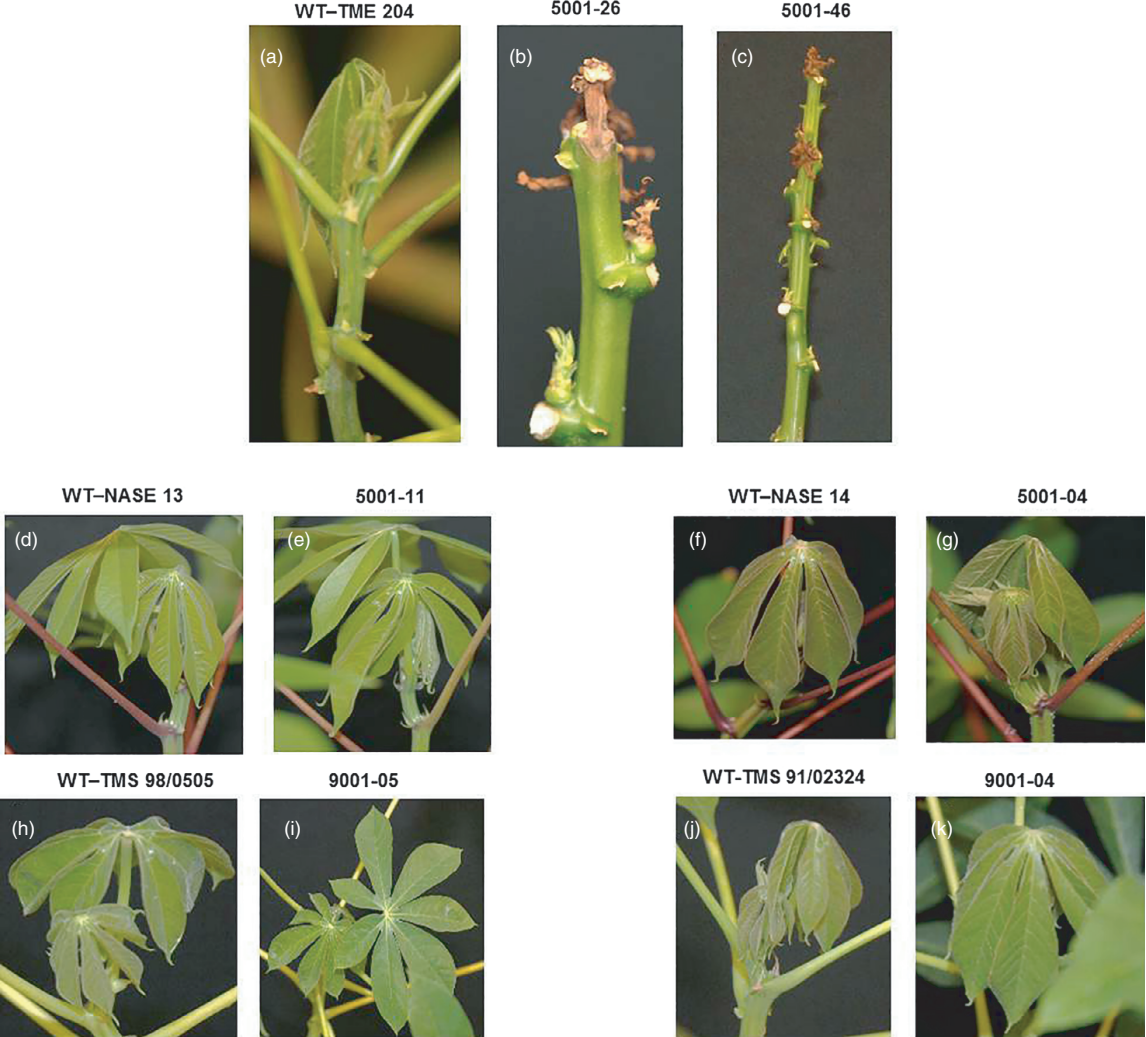
Transgenic p5001 and p9001 and respective non‐transgenic wild‐type control plants showing resistance to CMD when challenged with MeSPY1‐VIGS under greenhouse conditions. (a) WT‐TME 204 display asymptomatic leaves, while (b) CMD susceptible 5001‐26 and (c) CMD susceptible 5001‐46 show necrosis and death of the leaves and meristems. CMD resistant wild types (d) NASE 13, (f) NASE 14, (h) TMS 98/0505 and (j) TMS 91/02324 and respective transgenic plants (e) 5001‐NASE 13. (g) 5001‐NASE 14, (i) 5001‐TMS 98/0505, (k) 9001‐TMS 91/02324, showing resistance to CMD when challenged with MeSPY1‐VIGS geminivirus. Images were taken 14 days after inoculation.

In order to determine resistance to CMD, p5001 and p9001 transgenic events and respective wild‐type controls were challenged with MeSPY1‐VIGS. MeSPY1‐VIGS is a modification of the virulent K201 strain of East African cassava mosaic virus (Beyene *et al*, 2017b). Upon infection, MeSPY1‐VIGS causes necrosis and death in CMD susceptible cassava cultivars due to downregulation of the *SPINDLY* (*MeSPY*) gene, while CMD‐resistant varieties recover from infection and remain robust over a 3‐4‐week observation period. Non‐transgenic TME 204 controls displayed a transient reduction in growth followed by full recovery from symptoms (Figure 4a; Figure S5). In contrast, transgenic events 5001‐26 and 5001‐46 of TME 204 became necrotic and wilted 12–14 days after inoculation, leading to subsequent death of all plants (Figure 4b‐c; Figure S5). Non‐transgenic and transgenic lines of NASE 13, NASE 14, TMS 98/0505 and TMS 91/02324 demonstrated high‐level resistance to inoculation with MeSPY1‐VIGS (Figure 4d‐k). After challenge, plants resumed normal plant growth with 100% survival (Figure 4d‐k; Figure S5). An exception to this pattern was seen in a single NASE 14 transgenic event, line 5001‐41, in which 6 out of 7 clonal plants developed severe CMD symptoms and died in a manner similar to the known susceptible TME 204 events 5001‐26 and 5001‐46 (Figure S5b).

### Transgenic p9001 lines show elevated storage root iron and zinc concentrations

**Figure 5:**
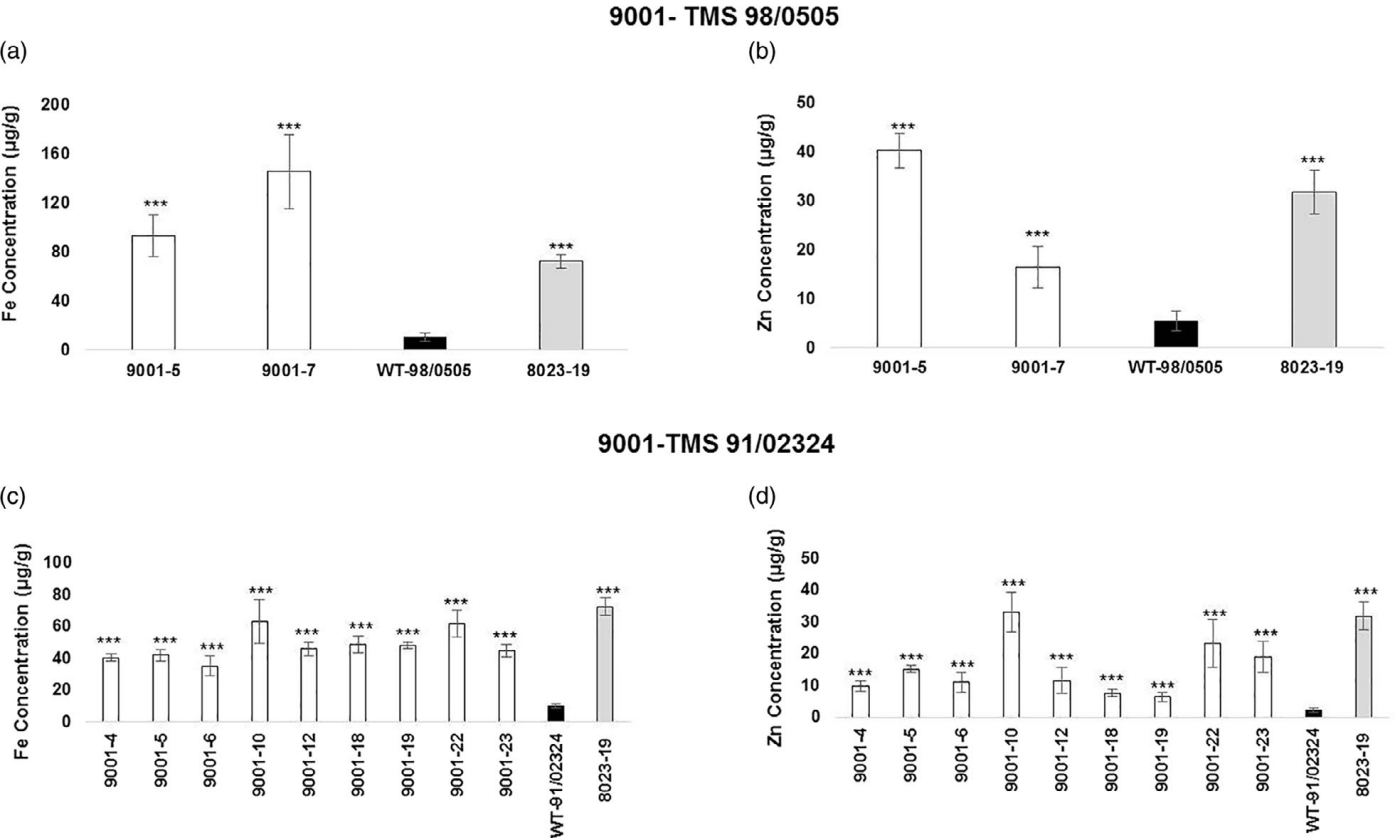
Storage root mineral concentrations of p9001 transgenic cassava plants grown under greenhouse conditions. (a) Fe and (b) Zn concentration of storage roots of p9001‐TMS 98/0505 transgenic cassava plants. (c) Fe concentration and (d) Zn concentration of storage roots of p9001–TMS 91/02324 transgenic cassava plants. Transgenic line 8023‐19 (TME 204) was used as a positive control (Narayanan *et al*., 2019). Values are means of four biological replicates. Error bars represent SD. *** denotes significant difference at *P* < 0.001.

Plants of cultivars TMS 98/0505 and TMS 91/02324 transgenic for p9001 expressing siRNAs against CBSD and mRNA for *IRT1* and *FER1* were established in the greenhouse. After 16 weeks growth, storage roots were harvested and mineral concentrations determined by Inductively coupled plasma optical emission spectrometry (ICP‐OES). Storage roots of transgenic TMS 98/0505 plants were found to have accumulated 8‐13 times higher iron and 2‐7 times higher zinc concentrations compared to the same tissues in non‐transgenic controls (Figure 5a‐b). Maximum iron accumulation reached 145 ± 30 µg/g dry wt. (DW) across replicates of TMS 98/0505 event 9001‐07 compared to 10 ± 3 µg/g DW in non‐transgenic controls. In the same cultivar, maximum zinc accumulation reached 40 ± 3 µg/g DW in the stacked event 9001‐05 compared to 5 ± 2 µg/g DW in the controls (Figure 5a‐b). In a similar manner, p9001 events of TMS 91/02324 accumulated 3–5 times higher iron concentrations and 2–13 times higher zinc concentration in their storage roots compared to the controls (Figure 5c‐d). Maximum iron accumulation reached 63 ± 13 µg/g DW across replicates of TMS 91/02324 event 9001‐10 compared to 10 ± 1 µg/g DW in non‐transgenic controls. In the same cultivar background, maximum zinc accumulation reached 33 ± 6 µg/g DW in line 9001‐10 compared to 2 ± 0.7 µg/g DW in the controls (Figure 5c‐d). Storage roots of some 9001 transgenic plants showed a minor, statistically significant increase in other minerals including manganese, cobalt, copper and cadmium concentrations compared to the non‐transgenic controls (Figures S6 and S7). No significant differences were seen for nickel (Fig. S6c, S7c). Importantly, and as reported previously (Narayanan *et al*., 2019), levels of all these metals remained below toxic levels and would not pose a threat to human health.

## Discussion

More than 190 million hectares of biotech crops were planted in 26 different countries in 2018 (ISAAA, 2018). To date, however, the benefits of crop biotechnology have had little impact on the wellbeing of smallholder farmers in developing economies. This is especially the case in sub‐Saharan Africa where hundreds of millions of people rely on vegetatively propagated crops such as cassava, banana/plantains, sweetpotato and yams for their caloric requirements and economic security. Delivering the benefits of gene modification to these populations requires that resources are specifically focused and adapted for staples such as cassava to produce enhanced products that meet farmer and consumer needs. To have the required effect at scale, this must also be addressed across geographic regions, with useful traits integrated into multiple, elite farmer‐preferred varieties and breeding lines. The present study describes progress towards delivering disease resistant and nutritionally enhanced cassava designed to meet the needs of smallholder farmers. This has been achieved in four agronomically important cassava cultivars grown and valued by farmers and consumers in East and West Africa.

RNAi‐mediated resistance to CBSD under greenhouse conditions and multi‐location field trials were reported in cv. TME 204 (Beyene *et al*., 2017a; Wagaba *et al*., 2016). Here we provide evidence that this technology can be applied across agronomically important cultivars. NASE 13 and NASE 14 are widely grown by farmers in Uganda (Abaca *et al*., 2013), around the Lake Victoria region and in Rwanda, while TMS 98/0505 and TMS 91/02324 are important in Nigeria (Ademiluyi and Mepba, 2013). Robust levels of resistance to CBSD were shown against inoculation with the virulent CBSV Naliendele strain (Figure 2). We provide evidence that expression of CP‐derived siRNAs against CBSV and UCBSV imparts very high resistance to CBSD, with virus titre remaining undetectable by qRT‐PCR diagnostics in the best performing lines of all four cultivars (Figure 1, Figure S4). CBSD has yet to reach West Africa, but has progressed westwards from its origin in East Africa to become widespread in Central Africa (Tomlinson *et al*., 2018). West African cassava production is therefore under imminent threat. Indeed, extreme vulnerability of at least some Nigerian cassava is shown here, with CBSV causing death in the majority of TMS 98/0505 plants within a few weeks of infection (Figure 2). Generation of resistant, farmer‐preferred West African planting materials represents an important contingency for Nigerian farmers against arrival of CBSD in the world’s largest cassava‐producing country.

Previous reports of African cassava modified by transgenesis and gene editing for farmer traits have been associated with the loss of inherent resistance to CMD (Mehta *et al*., 2019; Wagaba *et al*., 2016). Loss of resistance to a pathogen in this manner is unique in the literature and has been shown to result from the morphogenic systems used to regenerate cassava plantlets in culture (Beyene *et al*., 2016; Chauhan and Taylor, 2018). Robust resistance to the geminiviruses that cause CMD is essential for deployment of new cassava varieties due to the pandemic nature of this disease throughout Africa. Proof that plants of transgenically enhanced NASE 13, NASE 14, TMS 98/0505 and TMS 91/02324 retained CMD resistance (Figure 4) provides important evidence that biotechnology can be applied to cassava to produce valuable traits suitable for deployment to African farmers. It remains unknown why loss of resistance to CMD was observed in one transgenic event of NASE 14 (5001‐41), while other lines regenerated in this cultivar retained functional CMD resistance (Figure S5b). This highlights the importance of continued efforts to understand the biology behind loss of CMD resistance in CMD2‐type varieties, and the opportunity this presents to discover and fully understand the molecular mechanisms that impart resistance to geminiviruses in cassava.

Nutritional security for the global population is critical and could be improved through biofortification of staple food crops (Connorton and Balk, 2019). Populations that rely on starchy staples such as cassava are at risk from micronutrient deficiencies (Gegios *et al*., 2010). We provide evidence that mineral biofortification of cassava storage roots can be stacked with resistance to CBSD and CMD. Functionality of all three traits was demonstrated in TMS 98/0505 and TMS 91/02324, with iron and zinc levels elevated in storage roots of both cultivars (Figure 5). Previous studies under field conditions have shown that iron and zinc levels continue to accumulate in storage roots over time up to 12 months. The maximums of 145 and 40 µg/g DW achieved for iron and zinc here will continue to increase in field‐grown plants, with these essential minerals retained within the processed food products at levels that increase % EAR to nutritionally significantly levels (Narayanan *et al*., 2019). Cassava is the only dicotyledonous crop plant for which mineral biofortification has been reported in the harvested plant parts. Expanding this modification into additional cultivars demonstrates the potential of this technology to address nutritional needs in regions such as West Africa, while retaining resistance to CMD. Production of plants with elevated disease and mineral nutrition were technically more challenging to produce than for disease alone. This was apparent from the relatively low number of TMS 98/0505 plants produced for construct p9001 versus p5001, respectively (Table S2). A possible cultivar‐dependent response was also seen due to the higher numbers of stacked plants produced in TMS 91/02324 compared to TMS 95/0505 and is worth further investigation.

## Experimental procedures

### Vector construction and plant transformation

The two binary plasmid vectors p5001 (Beyene *et al*., 2017a) and p9001 were used to generate transgenic plants. p5001 was transformed into CMD resistant farmer‐preferred East African cassava cultivars NASE 13, NASE 14 and the Nigerian cultivar TMS 98/0505. p9001 (Figure S1) harbours the inverted repeat expression cassette CBSV‐CP and UCBSV‐CP present in p5001 plus *IRT1* and *FER1* for iron and zinc biofortification as described by Narayanan *et al*. (2019) with minor modifications. For the expression cassettes *IRT1* and *FER1*, homologous poly‐adenylation signals, *A14* 3’UTR for *IRT1* and patatin 3’UTR for *FER1* were used without additional 35S‐polyA nos terminator (Figure S1). The binary vector harbouring the *IRT1* and *FER1* plus *npt*II expression cassette for selection of transgenic plants was linearized with *AscI* to introduce the CBSV‐CP and UCBSV‐CP tandem inverted repeat expression cassette from p5001. The resulting p9001 construct was electroporated into *Agrobacterium tumefaciens* strain LBA4404 and was used to transform cassava cultivars TMS 98/0505 and TMS 91/02324. *Agrobacterium‐*mediated transformation of friable embryogenic callus (FEC) and regeneration of transgenic lines was performed as described earlier (Chauhan *et al*., 2015; Chauhan and Taylor, 2018).

### Plant establishment and growth in the greenhouse

All transgenic and control plants were micropropagated, transferred to potting soil (Conrad Fafard, Agawam, MA, USA), hardened and established in the greenhouse as described by Taylor *et al*. (2012). Plants were grown on the greenhouse bench at 30°C/27°C (day/night) with 70–95% relative humidity, watered with reverse osmosis water two or three times per day as required, and fertilized twice weekly with Jack’s professional fertilizer (JR Peters) at a rate of 100 µg/g DW (Taylor *et al*., 2012).

### Molecular characterization of transgenic plants

Transgenic in vitro plantlets were characterized for presence of the trait gene(s) and absence of vector backbone sequences using PCR and mRNA expression using RT‐qPCR, as described earlier using the primer pairs listed in Table S2 (Chauhan *et al*., 2015; Narayanan *et al*., 2019). Southern blots were performed to determine T‐DNA copy number following Chauhan *et al*. (2015). Twenty micrograms of genomic DNA from p5001 transgenic plants of cvs. NASE 13 and NASE 14 events were digested with 100 units of *NcoI* and *BstEII* (New England Biolabs), while p5001 TMS 98/0505 events were digested with 100 units of *BstEII* and *Bsu36I* (New England Biolabs). DNA extracted from p9001 lines of TMS 98/0505 and TMS 91/02324 was digested with 100 units of *Bsu36I* (New England Biolabs). All samples were digested overnight and subjected to electrophoresis on a 1% agarose gel. Fractionated DNA was transferred to a positively charged nylon membrane using downward capillary transfer in 0.4 m NaOH buffer (Sambrook *et al*., 2001). Membranes for Southern blots were hybridized to a 727 bp fragment of CBSV‐CP gene obtained from p5001 plasmid (Beyene *et al*., 2017a) using the primer pairs listed in Table S2. Hybridization of membrane‐bound DNA to DIG‐labelled probes was followed by washing and detection using CDP‐Star (Roche Applied Science, Indianapolis, IN) per manufacturer instructions. Signals were developed using HyperFilm ECL (GE‐Healthcare, Buckinghamshire, UK) as per manufacturer’s instructions.

### Detection of siRNAs by Northern blot analysis

Transgenic plants were analysed for the accumulation of CP‐derived siRNAs by Northern blotting. Total RNA was extracted from 100 mg fresh weight leaves collected from in vitro plantlets using TRIzol reagent (Ambion, Houston, TX, USA). Blotting, hybridization, UCBSV‐CP probe using SP6 polymerase and detection of siRNA were performed as described by Beyene *et al*. (2017a). Each Northern blot included control RNA extracted from plant lines 5001‐26 and 5001‐46 of cultivar TME 204. Signal strength was determined by scanning the blots, followed by quantification using ImageJ software v. 10.2 (Rasband, 1997–2019).

### Plant inoculation with CBSV

Nine transgenic lines of 5001‐NASE 13, six of 5001‐NASE 14, six of 5001‐TMS 98/0505, two of 9001‐TMS 98/0505 and nine transgenic lines of 9001‐TMS 91/02324 were assessed for resistance to CBSD. Between six and ten clonal plants per transgenic line were bud graft inoculated with CBSV at 7–10 weeks of age according to Wagaba *et al*. (2013). Inoculum was maintained in plants of cassava variety 60444 infected with CBSV isolate Naliendele (CBSV[TZ:Nal3‐1:07] (Mohammed *et al*., 2013, 2012). One plant per line was also set up as a non‐inoculated self‐graft control. Starting 10 days after inoculation, CBSD symptoms were scored visually on leaves and stems once per week using a 1‐5 scale (Hillocks and Thresh, 2000). Plant stems were cut back 6–7 weeks after graft inoculation to 5‐8 nodes above the graft union and newly emerging leaves and stem tissues were assessed for CBSD symptom development using the same 1‐5 scale. Between 12 and 14 weeks post‐inoculation, storage roots were harvested, washed and the peel removed. Storage roots were cut transversely along their length to produce slices of 1–2 cm thickness. Each slice was visually assessed and scored using a scale of 1‐5 for presence and severity of CBSD symptoms following the system described by Hillocks and Thresh (2000).

### Plant inoculation with geminivirus

Five transgenic lines each of 5001‐NASE 13, 5001‐NASE 14, 5001‐TMS 98/0505 and 9001‐TMS 91/02324 were assessed for resistance to CMD. A VIGS‐based screening method developed by Beyene *et al*. (2017b) was used to determine CMD resistance in transgenic and non‐transgenic control plants. Plasmid DNA‐A of *East African cassava mosaic virus* (EACMV‐K201) modified to carry *MeSPY1* (*Manihot esculenta* SPY) in the CP position and EACMV K201 DNA‐B component were co‐bombarded into young leaves of 6‐ to 8‐week‐old plants using a Helios Gene Gun (Bio‐Rad, Hercules, California, USA). Between six and eight plants of each transgenic line, non‐transgenic controls were inoculated in this manner. Scoring of plants to assess shoot‐tip necrosis and death was performed starting 7–14 days after inoculation according to Beyene *et al*. (2016).

### CBSV level determination by RT‐qPCR

Total RNA was isolated from 50–100 mg dry weight storage roots using the Fruitmate kit (Fruitmate^™^, Takara, Shiga, Japan) per manufacturer instructions, followed by on‐column DNAse I treatment (Sigma Aldrich, St. Louis, MO, USA). One microgram of total RNA was reverse transcribed using the SuperScript® III‐First‐Strand Synthesis System (Invitrogen, Waltham, MA, USA). CBSV titre in transgenic and non‐transgenic control plants were quantified by RT‐qPCR (Ogwok *et al*., 2015) using the primer pairs listed in Table S2, plus 10 ng reverse transcriptase template and SsoAdvanced™ Universal SYBR® Green Supermix (Bio‐Rad laboratories Inc., Hercules, CA). The endogenous cassava gene *PP2A* was used as an internal control (Moreno *et al*., 2011). PCR cycling conditions comprised an initial denaturation holding stage at 95°C for 30 s, followed by 40 cycles of cycling stage at 95°C for 5 s, 61°C for 30 s, melt curve stage from 65°C to 95°C with 0.5 increments for 5 s, followed by final extension at 95°C for 5 min. For each sample, reactions were set up in triplicates and three biological plants per independent event analysed to ensure reproducibility. Quantification of the relative transcript levels was performed using the comparative C_T_ (threshold cycle) method (Livak and Schmittgen, 2001).

### Measurement of mineral concentrations

Two transgenic lines of 9001‐TMS 98/0505 and nine transgenic lines of 9001‐TMS 91/02324 were assessed for accumulation of minerals within their storage roots. Storage root tissues were harvested from greenhouse‐grown transgenic and non‐transgenic control plants at 16 weeks after planting in soil and dried for 48 h in a 60°C oven. Homogenized dried tissues were processed for mineral analysis by weighing samples (approximately 500 mg DW each) into Teflon digestion vessels along with 10 mL of ultrapure nitric acid and an iridium internal standard (50 µg). Samples were heated in sealed, pressurized Teflon vessels with a microwave digestion system (MARS6; CEM Corporation, Matthews, NC) per the manufacturer’s instructions. The iridium standard was used to correct for any loss of volume during the microwave digestion. After cooling, the digestates were diluted 30‐fold with distilled water and elemental concentrations determined by inductively coupled plasma optical emission spectrometry (ICP–OES) (iCAP 7000; Thermo Electron North America LLC, Madison, WI, USA). The instrument was calibrated daily with certified standards. Peach‐leaf standards (SRM 1547 A; National Institute of Standards and Technology, USA) were digested and analysed along with each run of experimental samples to verify reliability of the procedures and analytical measurements; all values for peach‐leaf standards were within their certified range. Four biological replicates were processed for each sample.

### Statistical analysis

Greenhouse shoot and root scoring of CBSD and mineral concentration data collected from storage root tissues were subjected to one‐way analysis of variance (ANOVA), and where significant mean separation was done by Dunnett test using Minitab 17 Statistical Software (Minitab *et al*., 2013, 2012).

## Conflict of interests

This work was supported by the Bill & Melinda Gates Foundation (BMGF) through the Global Challenges for Global Health Program (Grant no: OPP1152626), and the US Department of Agriculture, Agricultural Research Service, Project no. 008‐3060‐253 (to M.A.G.). The contents of this publication do not necessarily reflect the views or policies of the US Department of Agriculture nor does mention of trade names, commercial products or organizations imply endorsement by the US Government. BMGF had no role in the study design, collection, analysis or interpretation of data, in the writing of the report or the decision to submit the article for publication. This work was funded in whole (or part) by the United States Agency for International Development (USAID) under Agreement EDH‐A‐00‐09‐00010‐00 as part of ‘Feed the Future Innovation Laboratory’. Any opinions, findings, conclusions or recommendations expressed here are those of the authors alone.

## Authors’ contributions

N.N., G.B. and N.J.T. conceived and designed experiments. G.B. designed and constructed vectors for transformation. R.D.C., and N.J.T. generated the transgenic plants. N.N., and G.B. performed molecular and trait analysis. M.A.G., and N.N. performed mineral analysis. N.N. and G.B. analysed datasets. N.N. and N.J.T. wrote manuscript.

## Supporting information


**Figure S1** Schematic representation of stacked T‐DNA construct p9001 consisting of inverted repeats of coat protein sequences of CBSV and UCBSV found in p5001 (Beyene et al. 2017) and IRT1 and FER genes for nutritional enhancement.
**Figure S2** Quantitative expression of (a) *AtIRT1* and (b) *AtFER1* in p9001‐TMS 98/0505 and p9001‐TMS 91/02324 transgenic cassava plants.
**Figure S3** Southern blot analysis of independent plant lines transgenic for constructs p5001 and p9001 in cassava cultivars (a) NASE 13 and NASE 14, (b) TMS 98/0505, (c) TMS 98/0505 and TMS 91/02324. *Lane*‐marker restricted dig ladder, WT‐wild type.
**Figure S4** qRT‐PCR detection of cassava brown streak virus in storage roots of transgenic p5001 and p9001 plant lines.
**Figure S5** Survival rate of MeSPY1‐VIGS challenged cassava.
**Figure S6** Mineral concentrations within storage roots harvested from p9001‐TMS 98/0505 transgenic cassava plants grown under greenhouse conditions.
**Figure S7** Mineral concentrations within storage roots harvested from p9001‐TMS 91/02324 transgenic cassava plants grown under greenhouse conditions.

**Table S1** Production and analysis of transgenic cassava plants.
**Table S2** Primers used for analysis of transgenic plants.
