# Supplementary material for: Stacking disease resistance and mineral biofortification in cassava varieties to enhance yields and consumer health
Source: Plant Biotechnol J. 2020 Dec 10;19(4):844–54. doi: 10.1111/pbi.13511 (PMC8051606; doi:10.1111/pbi.13511)
Supplement: Supplementary file 1 — Figure S1 Schematic representation of stacked T‐DNA construct p9001 consisting of inverted repeats of coat protein sequences of CBSV and UCBSV found in p5001 (Beyene et al. 2017) and IRT1 and FER genes for nutritional enhancement. Figure S2 Quantitative expression of (a) AtIRT1 and (b) AtFER1 in p9001‐TMS 98/0505 and p9001‐TMS 91/02324 transgenic cassava plants. Figure S3 Southern blot analysis of independent plant lines transgenic for constructs p5001 and p9001 in cassava cultivars (a) NASE 13 and NASE 14, (b) TMS 98/0505, (c) TMS 98/0505 and TMS 91/02324. Lane‐marker restricted dig ladder, WT‐wild type. Figure S4 qRT‐PCR detection of cassava brown streak virus in storage roots of transgenic p5001 and p9001 plant lines. Figure S5 Survival rate of MeSPY1‐VIGS challenged cassava. Figure S6 Mineral concentrations within storage roots harvested from p9001‐TMS 98/0505 transgenic cassava plants grown under greenhouse conditions. Figure S7 Mineral concentrations within storage roots harvested from p9001‐TMS 91/02324 transgenic cassava plants grown under greenhouse conditions. [file PBI-19-844-s001.pdf]

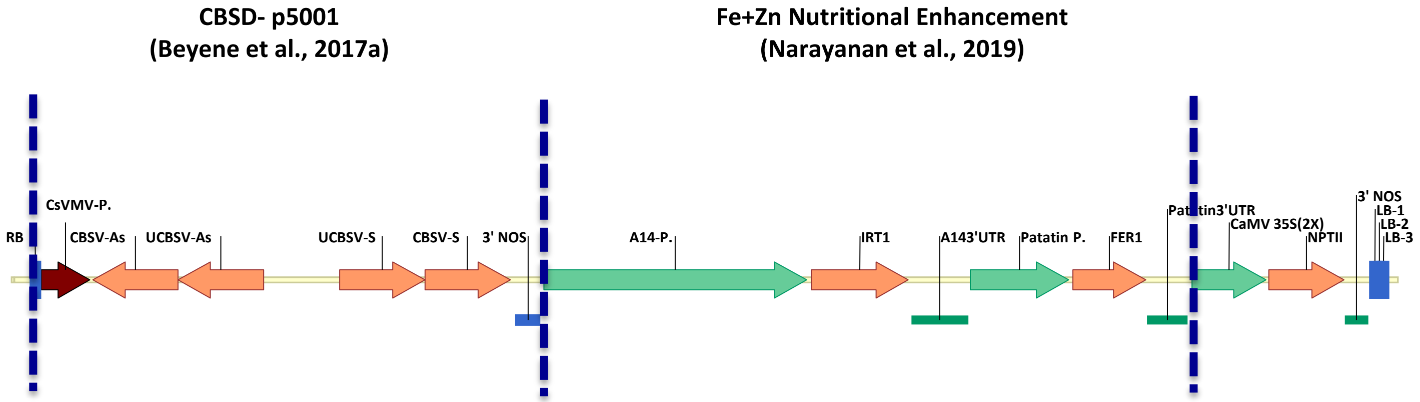

Supplemental Figure 1 Schematic representation of stacked T-DNA construct p9001 consisting of inverted repeats of coat protein sequences of CBSV and UCBSV found in p5001 (Beyene et al. 2017) and *IRT1* and *FER* genes for nutritional enhancement. RB and LB symbolizes right and left borders of the T-DNA respectively. CsVMV-P: Cassava vein mosaic virus promoter, CBSV: Cassava brown streak virus, UCBSV: Ugandan cassava brown streak virus, As: anti-sense strand, s: sense strand, A14: *Arabidopsis* root epidermal promoter, *AtIRT1*: iron regulated transporter from *Arabidopsis*, 3' *A14*: 3'UTR from A14, patatin: promoter from potato, *AtFER1*: ferritin storage protein from *Arabidopsis, 3' pat*: 3' UTR from patatin, , CaMV 35S (2X): Cauliflower mosaic virus 35S promoter, NPTII: Neomycin phosphotransferase gene, 3'Nos: 3' UTR from *Agrobacterium*.

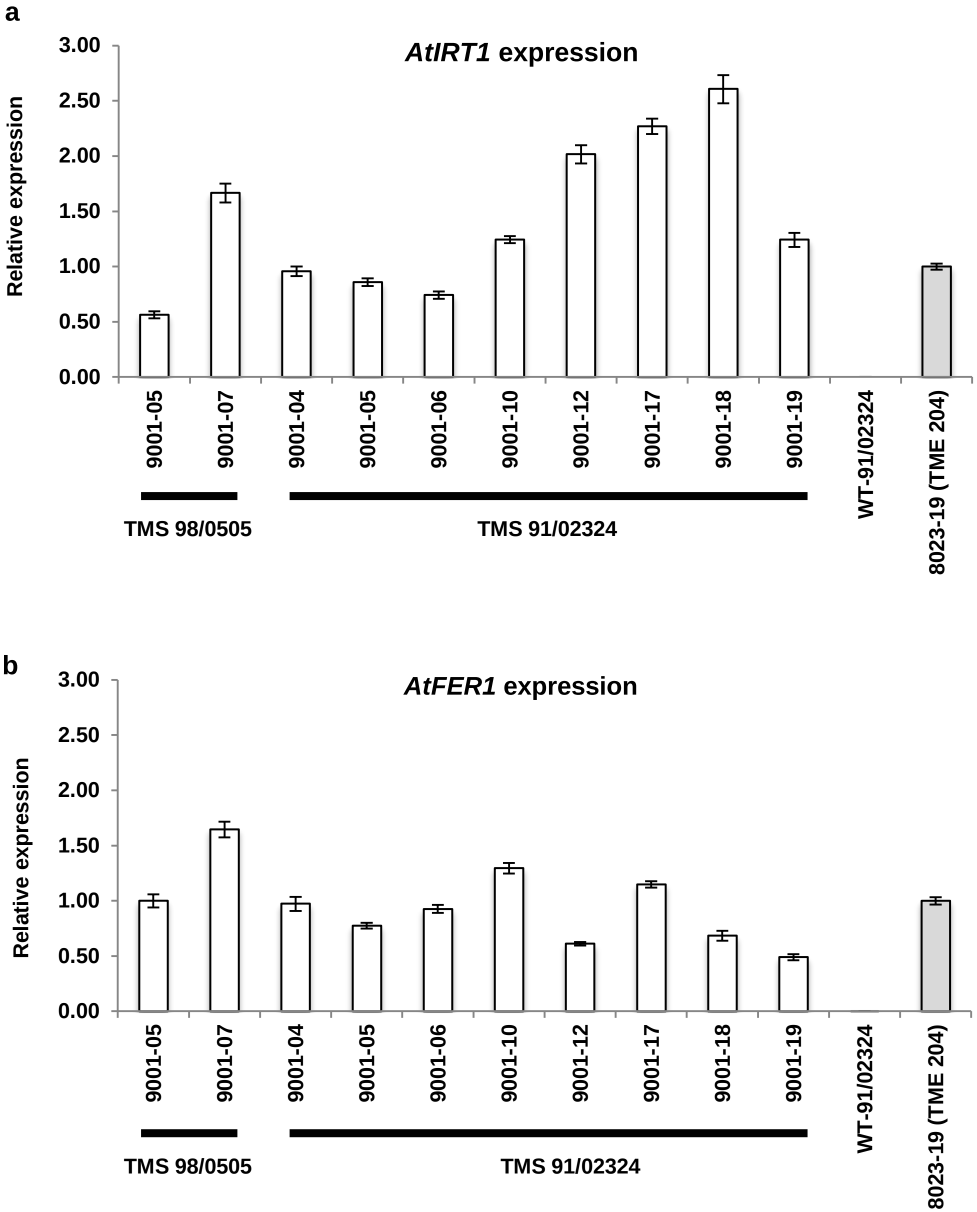

Supplementary Figure 2 Quantitative expression of **(a)** *AtIRT1* and **(b)** *AtFER1* in p9001-TMS 98/0505 and p9001-TMS 91/02324 transgenic cassava plants. Leaf tissues were collected from 16-week-old plants grown in the greenhouse. Expression was compared and normalized to protein phosphatase 2 (pp2A). Transgenic line 8023-19 (TME 204) was used a positive control (Narayanan et al., 2019). Expression value of 8023-19 was adjusted to a value of 1, and all other expression values were expressed relative to this line. Values are means of four biological and three technical replicates. Error bars represent SD.

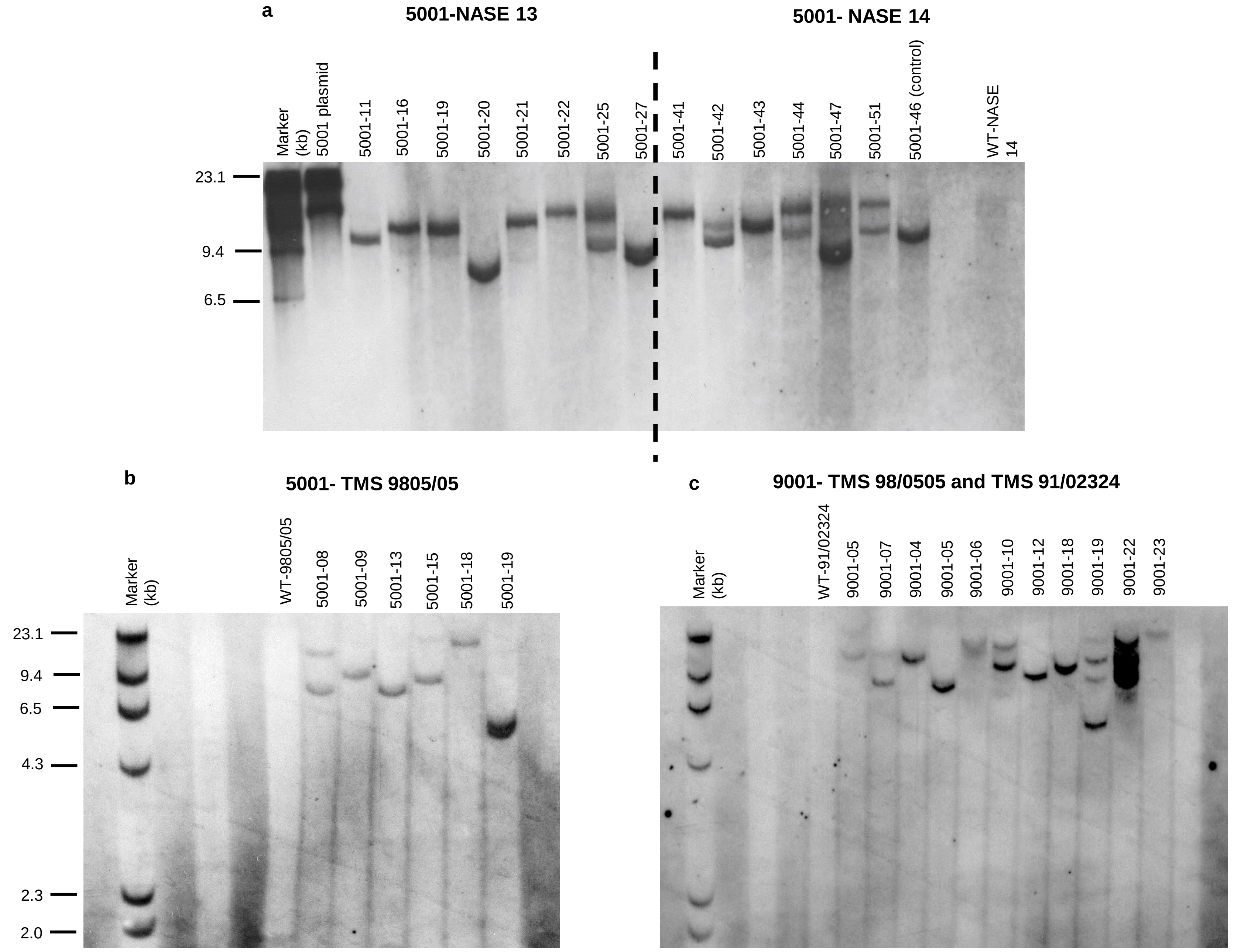

Supplemental Figure 3 Southern blot analysis of independent plant lines transgenic for constructs p5001 and p9001 in cassava cultivars **(a)** NASE 13 and NASE 14, **(b)** TMS 98/0505, **(c)** TMS 98/0505 and TMS 91/02324. *Lane* - marker restricted dig ladder, WT-wild type.

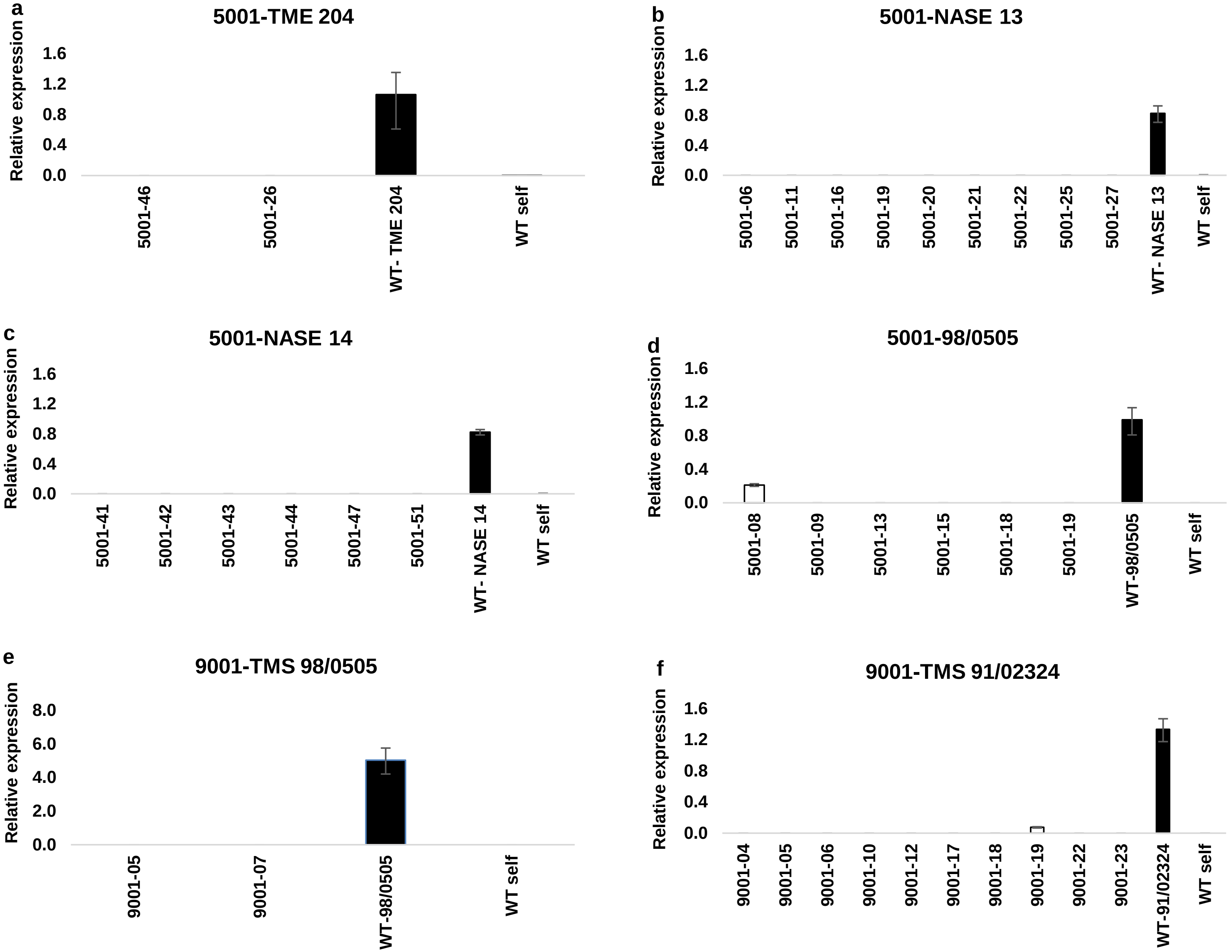

Supplemental Figure 4 qRT-PCR detection of cassava brown streak virus in storage roots of transgenic p5001 and p9001 plant lines. Quantitative expression of CBSV in independent plant lines of **(a)** TME 204, **(b)** p5001-NASE 13, **(c)** p5001- NASE 14, **(d)** p5001-TMS 98/0505, **(e)** p9001-TMS 98/0505 and **(f)** p9001-TMS 91/02324. Reactions were set up in triplicates with three biological plants per independent event. Quantification of the relative transcript levels was performed using the comparative C_T (threshold cycle) method. Storage root tissues were collected from storage roots 12-14 weeks after bud graft inoculation with the Naliendele isolate of CBSV. Expression was compared and normalized to protein phosphatase 2 (*pp2A*). Error bars represent SD.

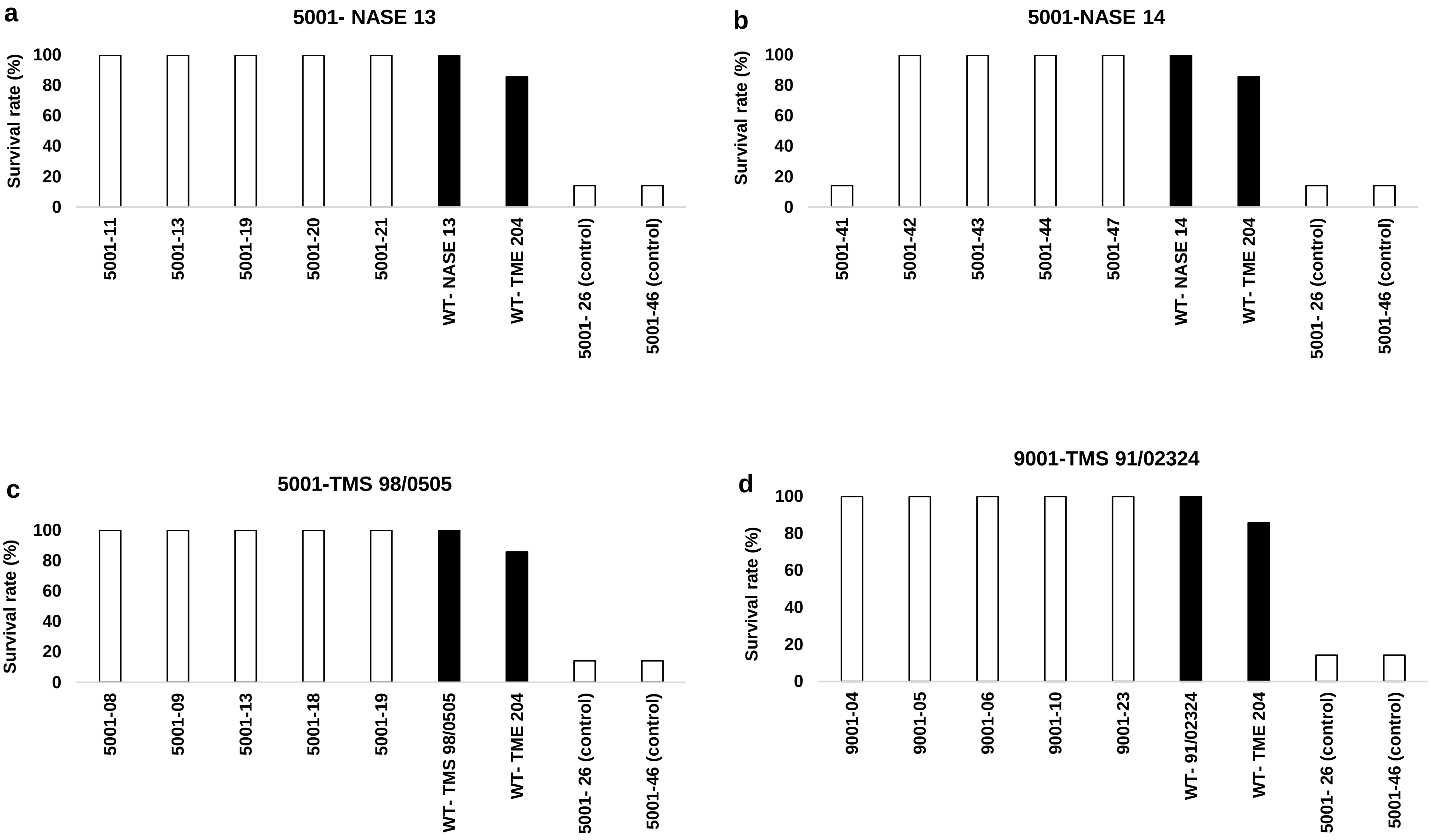

Supplemental Figure 5 Survival rate of MeSPY1-VIGS challenged cassava. Four-week old transgenic cassava lines **(a)** p5001-NASE 13, **(b)** p5001- NASE 14, **(c)** p5001-TMS 98/0505 and **(d)** p9001-TMS 91/02324 plus wild type were challenged with MeSPY1-VIGS and evaluated for survival at 5 weeks after challenge. Six to seven plants were challenged per cultivar/line. Plants were considered dead if the shoot-tip had completely died, and leaves had defoliated or were wilting and defoliating at evaluation time. Survival is expressed as a percent of the total number of challenged plants per transgenic event.

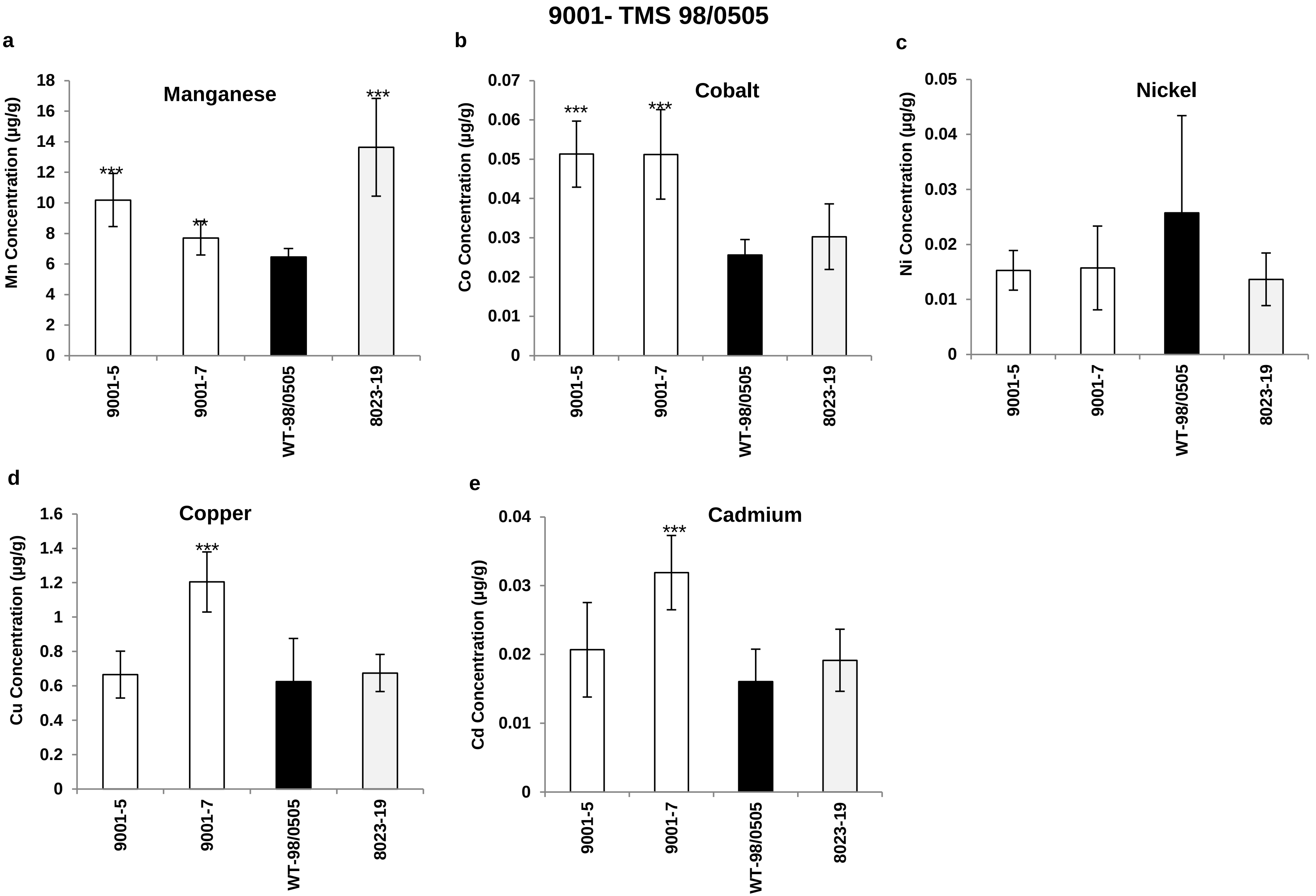

Supplemental Figure 6 Mineral concentrations within storage roots harvested from p9001-TMS 98/0505 transgenic cassava plants grown under greenhouse conditions. **(a)** Mn, **(b)** Co, **(c)** Ni, **(d)** Cu and **(e)** Cd. Transgenic line 8023-19 (TME 204) was used as a positive control (Narayanan et al., 2019). Values are means of four biological replicates. Error bars represent SD. *** denotes significant difference at *p<0.001*.

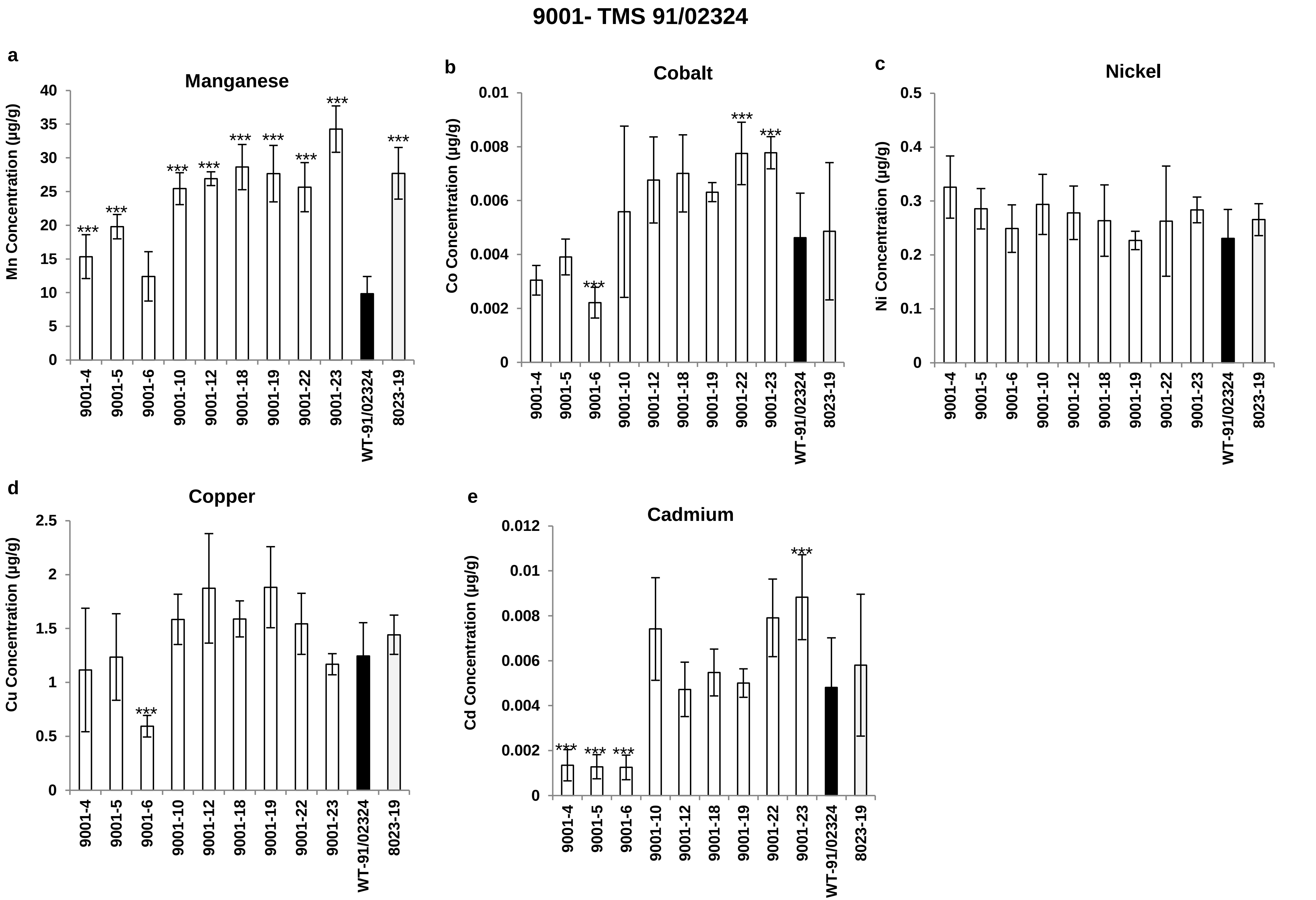

Supplemental Figure 7 Mineral concentrations within storage roots harvested from p9001-TMS 91/02324 transgenic cassava plants grown under greenhouse conditions. **(a)** Mn, **(b)** Co, **(c)** Ni, **(d)** Cu and **(e)** Cd. Transgenic line 8023-19 (TME 204) was used as a positive control (Narayanan et al., 2019). Values are means of four biological replicates. Error bars represent SD. *** denotes significant difference at *p<0.001*.
