# Supplementary material for: Stacking disease resistance and mineral biofortification in cassava varieties to enhance yields and consumer health
Source: Plant Biotechnol J. 2020 Dec 10;19(4):844–54. doi: 10.1111/pbi.13511 (PMC8051606; doi:10.1111/pbi.13511)
Supplement: Supplementary file 2 — Table S1 Production and analysis of transgenic cassava plants. Table S2 Primers used for analysis of transgenic plants. [file PBI-19-844-s002.docx]

**Supplemental Table 1** Production and analysis of transgenic cassava plants

| Cultivar | Construct | Gene(s) of interest | # independent plant lines regenerated | # lines analyzed by PCR | # lines positive for transgenes | # lines free of VBB sequences |
| --- | --- | --- | --- | --- | --- | --- |
|  |  |  |  |  |  |  |
| NASE 13 | 5001 | UCBSV-CP/CBSV-CP | 155 | 121 | 93 | 50 |
| NASE 14 | 5001 | UCBSV-CP/CBSV-CP | 259 | 259 | 100 | 45 |
| TMS 98/0505 | 5001 | UCBSV-CP/CBSV-CP | 153 | 147 | 139 | 58 |
| TMS 98/0505 | 9001 | UCBSV-CP/CBSV-CP+IRT1+FER | 51 | 50 | 25 | 14 |
| TMS 91/02324 | 9001 | UCBSV-CP/CBSV-CP +IRT1+FER | 56 | 55 | 45 | 28 |
|  |  |  |  |  |  |  |

VBB – vector backbone. Primers used to screen transgenic events are shown in Supplemental Table 2.

**Supplemental Table 2** Primers used for analysis of transgenic plants

| **Primer number** | **Sequence** | **Target gene** | **Purpose** |
| --- | --- | --- | --- |
| 524 | GCTTCGGACTTGTAAGATTCATCAGA | IRT1-F | PCR |
| 525 | TCATTCTGTTGTGATCGGACTTTCC | IRT1-R | PCR |
| 526 | GAGGTTGAAATACTTAAATGCGTGC | FER-F | PCR |
| 232 | ACCGGAGTCGTGTTCCAGCCTT | FER-R | PCR |
| 1072 | GATGTCATACAGAATTTTCTAGCG | CBSD-F | PCR |
| 1073 | GGCTTTGTGACAATTGTGCT | CBSD-R | PCR |
| 381 | GCAGGAGACATTCCTTCCGTATCTT | - | Vector back bone |
| 382 | CGATGATTAGGGAACGCTCGAACT | - | Vector back bone |
| 1058 | TCCATCAGCTTCGGACTTGTAAGA | IRT1-F | RT-qPCR |
| 1059 | GGAGGAATGTCCATTATCGCCA | IRT1-R | RT-qPCR |
| 1060 | GAGGTTGAAATACTTAAATGCGTGC | FER-F | RT-qPCR |
| 1061 | TAAGGATGATCGGCAAAGGCCA | FER-R | RT-qPCR |
| 956 | GCTGAGATGATGGCTGAGGAGAA | CBSV-F | RT-qPCR |
| 957 | CGCCCTTTGCAAAGCTGAAATAAC | CBSV-R | RT-qPCR |
| 837 | TGCAAGGCTCACACTTTCATC | PP2A-F | RT-qPCR |
| 838 | CTGAGCGTAAAGCAGGGAAG | PP2A-R | RT-qPCR |
| 13 | CAAACGGACAAAGGAAAAGC | CBSV-CP-F | Probe-Southern blot |
| 16 | GGCTTTGTGACAATTGTGCTTC | CBSV-CP-R | Probe- Southern blot |
